# Combination of the toll like receptor agonist and α-Galactosylceramide as an efficient adjuvant for cancer vaccine

**DOI:** 10.1186/s12929-016-0238-3

**Published:** 2016-01-25

**Authors:** Fateme Gableh, Mohsen Saeidi, Shaghayegh Hemati, Kasra Hamdi, Hoorieh Soleimanjahi, Ali Gorji, Amir Ghaemi

**Affiliations:** Infectious Diseases Research Center, Department of Microbiology, Golestan University of Medical Sciences, POBox: 49175-1141, Gorgan, Iran; Stem Cell Research Center, Golestan University of Medical Sciences, Gorgan, Iran; Guilan Science and Research Branch, Islamic Azad University, Rasht, Iran; Department of microbiology, Islamic Azad University, Shiraz branch, Shiraz, Iran; Department of Virology, Faculty of Medical Sciences, Tarbiat Modares University, Tehran, Iran; Shefa Neuroscience Research Center, Tehran, Iran; Epilepsy Research Center, Institut für Physiologie I, Westfälische Wilhelms-Universität Münster, Robert-Koch-Strasse, Münster, Germany; Klinik und Poliklinik für Neurochirurgie, Department of Neurology, Westfälische Wilhelms-Universität Münster, Münster, Germany; Department of Virology, Pasteur Institute of Iran, Tehran, Iran

**Keywords:** Human Papilloma Virus, DNA vaccine, E7, Adjuvant combination, α-Galactosylceramide, MPL

## Abstract

**Background:**

DNA vaccines have emerged as an attractive approach for the generation of cytotoxic T lymphocytes (CTL). In our previous study, we found That Toll like receptor (TLR) ligands are promising candidates for the development of novel adjuvants for DNA vaccine. To improve the efficacy of DNA vaccine directed against human papillomavirus (HPV) tumors, we evaluated whether co-administration of a TLR4 ligand, monophosphoryl lipid A (MPL), and Natural Killer T Cell Ligand α-Galactosylceramide(α-GalCer) adjuvants with DNA vaccine would influence the anti-tumor efficacy of DNA vaccinations.

**Methods:**

We investigated the effectiveness of α-GalCer and MPL combination as an adjuvant with an HPV-16 E7 DNA vaccine to enhance antitumor immune responses.

**Results:**

By using adjuvant combination for a DNA vaccine, we found that the levels of lymphocyte proliferation, CTL activity, IFN- γ, IL-4 and IL-12 responses, and tumor protection against TC-1 cells were significantly increased compared to the DNA vaccine with individual adjuvants.

In addition, inhibition of IL-18 signaling during vaccination decreased IFN-γ responses and tumor protection, and that this inhibition suggested stimulatory role of IL-18 in adjuvant effects of α-GalCer and MPL combination.

**Conclusion:**

The strong adjuvanticity associated with α-GalCer/MPL combination may to be an important tool in the development of novel and strong cancer immunotherapy.

## Background

Cervical cancer is the second most common cause of malignancies in women worldwide, and the oncogenic activity of the E7 protein that is expressed by high-risk human papilloma viruses (HPVs), and in particular by the HPV-16 genotype, has a pivotal role in anogenital tumors [1, 2].

Several lines of evidence suggest that cell-mediated immunity is important in controlling both HPV infection and HPV-associated neoplasms [3]. Therefore, vaccines or immunotherapies targeting E7 protein may provide the opportunity to prevent and treat HPV-associated malignancies [4, 5].

DNA vaccines can induce both humoral and cellular immune responses. The clinical benefits of DNA vaccine are low cost, vaccine stability, high productivity, and easy modification of antigen in comparison with traditional protein vaccines. Indeed, DNA vaccines have shown remarkable success in most animal studies and clinical trials in humans [6, 7].

However, low immunogenicity has proved a significant obstacle to efficacy for DNA vaccines, especially in higher primates and humans [8]. To date, various approaches have been taken to enhance the potency of such vaccines [9, 10].

Among the strategies to enhance DNA vaccination, the use of adjuvant appears to be particularly promising. Adjuvants are powerful substances to enhance the immunogenicity and boost the protective immunity elicited by vaccines [11–13]. Toll-like receptors (TLRs) play an important role in the innate recognition of pathogen-associated molecular patterns (PAMPs) and initiation of immune responses in dendritic cells (DCs) [14]. TLR ligands have therefore emerged as potential vaccine adjuvants, particularly in the context of peptide, protein, and DNA vaccines [4, 15]. Single TLR agonists are currently being developed and utilized for vaccination and tumor immunotherapy. Certain combinations of adjuvant molecules could have advantages over use of a single agonist, as there is evidence that the use of individual TLR agonists in combination with non-TLR agonists may more efficiently generating or effectively directing immune responses [16]. Among the TLR agonists, TLR4 agonists hold particular promise effect as potential vaccine adjuvants for use in tumors immunotherapy [17]. The first TLR4 agonist approved for human use as a vaccine adjuvant is a chemically-modified natural lipid A product derived from the LPS of Salmonella Minnesota R595 and known as monophosphoryl lipid A (MPL) [18]. MPL is an effective adjuvant in prophylactic and therapeutic vaccines and presents an outstanding safety in humans [19, 20].

α-Galactosylceramide (α-GalCer) represents another group of compounds that has shown to have an strong effect as vaccine adjuvant for immunotherapy of tumors [21]. α-GalCer is a glycolipid that has been identified as a ligand recognized by Va14 natural killer T (iNKT) cells. A key feature of iNKT cells is the expression of a single invariant Va14 antigen receptor that recognizes glycolipid antigens in mice [22]. Several studies have reported that α-GalCer may be used as a systemically delivered vaccine adjuvant for the induction of potent natural killer cell-dependent anti-tumor cytotoxic responses [23]. α-GalCer enhanced anti-tumor immunity in mice when administered in combination with various types of vaccines [10, 24].

It has been shown that the simultaneous induction of various immune targets by combination of adjuvants could generate a more effective and longer lasting antitumor immune response; therefore, in the present study we hypothesized that the combination of NKT cell antigen α-GalCer and MPL may potentiate their antitumor effects for immunotherapy of cancer. We investigated the combined adjuvant activity of α-GalCer and MPL for DNA vaccine-induced protective and antitumor immunity against S.C. TC-1 tumors in C57BL/6 mice.

## Methods

### Animals

Six to eight week-old female C57BL/6 mice were purchased from the Pasteur Institute (Karaj, Iran) and kept in the laboratory animal facility of Golestan University of medical sciences. All animals were fed with enough food and water to pass adaptation period, and treated with 6.00 to18.00-hour light/dark cycle. Approved protocols were applied to all animal experiments with consideration of recommendations for the accurate use and care of laboratory animals by the ethical commission of Golestan University.

### Adjuvants and blocking antibodies

The iNKT cell ligand α-GalCer was supplied by Enzo Life Sciences and was solubilized in 150 mM NaCl, 0.5 % Tween 20, referred to as vehicle.

Pure monophosphoryl lipid A (Synthetic lipid A from *E. coli*, serotype R515 from Invivogen) as TLR4 agonist were dissolved in PBS for a final concentration of 1 μg/ml. The doses used were as follows: 1 μg of GalCer (or equivalent PBS-diluted vehicle solution) and 25 μg of MPL per subcutaneous (S.C.) injection.

Blockade of IL-18 was carried out with 100 mg of recombinant murine anti-mouse IL-18 clone 93–10 (R&D Systems) or an isotype matched control.

### DNA vaccine preparation

The generation of pcDNA3.1-E7 has been described previously [25]. Plasmid constructs were confirmed by DNA sequencing and expression. Amplification and purification of DNA were previously described. Stocks of endotoxin free DNA vaccine plasmids and vector control plasmid (pcDNA3.1) in 0.1 M PBS were prepared for in-vivo immunization studies using the EndoFree® Plasmid Maxi Kit (Qiagen, Hilden, Germany) and dissolved in endotoxin-free Tris-EDTA (Sigma, St. Louis, MO).

### Cell lines and culture medium

TC-1, (part of the Johns Hopkins Special Collection) was derived from primary epithelial cells of C57BL/6 mice co-transformed with HPV16 E6 and E7 and activated c-Ha-ras oncogene. TC-1 cell line (HPV-16 E7^+^) was used as a tumor model in an H-2b murine system. The EL4 cells, a mouse lymphoma cell line derived from C57BL/6- Ly5.2 mice were obtained from the National Cell Bank of Iran (NCBI, Pasteur Institute, Tehran). TC-1 and EL4 cell lines were grown in RPMI 1640 (GIBCO, UK), supplemented with 10 % FBS, 2 mM L-glutamine, 25 mM HEPES, penicillin (100 U/ml), streptomycin (100 μg/ml) and G418 0.4 mg/ml at 37 °^C^ with 5 % CO_2_.

### In vivo tumor treatment experiment using TC-1 tumor cells

C57BL/6 mice were challenged with 2 × 10^5^ TC-1 tumor cells/mouse by S.C. injection in the right flank in 0.2 ml of PBS using a 25G needle for the in vivo tumor treatment experiment.

After 1 week, the C57BL/6 were immunized three times by S.C. injection of DNA vaccine encoding HPV-16 E7 (E7 DNA vaccine, 100 μg in 100 μl) with or without adjuvants of α-GalCer (1 μg), MPL (25 μg) or combinations of α-GalCer (0.5 μg) and MPL (12.5 μg) thrice at 7-day intervals. As a control, mice were given pcDNA3, PBS, 1 μg α-GalCer or 25 μg MPL alone.

Subcutaneous tumor volume was estimated according to Carlsson’s formula [25]. Hence, the largest (a) and the smallest (b) superficial diameters of the tumor were measured in a blinded, coded fashion twice a week and then the volume (V) of the tumor was calculated (*V* = a × b × b/2). The tumor volume was monitored up to 6 weeks after tumor challenge.

Statistical analysis was performed using Student’s *t* test. All values were expressed as means ± S.D. Three mice per group were sacrificed 1 week following the third immunization and the spleens were removed aseptically, and then cell proliferation, cytolytic activity and cytokine secretion were assayed. Six mice were also used for IL-18 blockade experiment.

All tests were performed in triplicate for each mouse. Results are representative of three independent experiments.

### Preparation of splenocytes

Mice were sacrificed and spleens removed using aseptic technique. Spleens were removed, and the resulting single-cell suspensions were pelleted, and the red blood cells were lysed by using a lysis buffer (0.15 M NH_4_Cl; 1 mM KHCO_3_; 0.1 mM Na_2_EDTA; pH 7.2). Cells were then washed and counted. Splenocytes were resuspended in RPMI 1640 supplemented with 10 % FBS, 1 % L-glutamine, 1 % HEPES, 0.1 % 2-mercaptoethanol and 0.1 % penicillin/streptomycin (all from Gibco).

### Cytotoxicity assay

One week after last immunization, the mice (three mice of each group) were sacrificed and their splenocytes were isolated. For each sample obtained from individual mice, single-cell suspensions of mononuclear cells (used as the effector cells) were cultured in RPMI 1640 medium with washed EL4 target cells (a mouse lymphoma cell line derived from C57BL/6 (MHC-H2b); ATCC TIB-39, from the National Cell Bank of Iran (NCBI, Pasteur Institute, Tehran)) at various effector-to-target cell (E/T) ratios (25:1, 50:1, 100:1) and in 96-well flat-bottom plates for 4 h in phenol red-free RPMI 1640 containing 3 % FBS.

For preparation of the target cells, EL4 cells were stimulated with E7-specific H-2Db CTL epitope at a concentration of 1 μg/ml and then incubated for 4 h. After centrifugation, the supernatants (50 μl/well) were transferred into the 96-well flat-bottom plates, and lysis of target cells were determined by measuring lactate dehydrogenase (LDH) release using a LDH cytotoxicity detection kit according to the procedures stated by the manufacturer (Takara Company, Shiga, Japan). Several controls were used for the cytotoxicity assay.

The ‘target maximum’ was the total LDH released from the target cells, and all EL4 cells were lysed by medium containing 1 % Triton X-100. The ‘target spontaneous’ was the natural release of LDH from the target cells, which was obtained by adding EL4 cells only to the assay medium. The ‘T cell control’ was used to measure the natural release of LDH from T cells and was obtained by adding the different ratios of T cells only to the assay medium.

For all samples, including the controls, the assay was performed in triplicate. The LDH-mediated conversion of tetrazolium salt into a red formazan product was measured at 490 nm after incubation at room temperature for 30 min. The percentage of specific cytolysis was determined by the following formula:

$$ \mathrm{Cytotoxicity} = \left[\left(\mathrm{experimental}\ \mathrm{value} - \mathrm{effector}\ \mathrm{spontaneous}\right) - \frac{Low\  control}{High\  control- Low\  control}\right] \times 100 $$

### Lymphocyte proliferation assay

One week after the third immunization, the splenocytes at a concentration of 2 × 10^5^ cells/well were cultured in 96-well flat-bottom culture plates (NalgeNunc International, Denmark) in the presence of E7-specific H-2Db CTL epitope at a concentration of 1 μg/ml, unspecific mitogen ConA (2 μg/ml) or media at 37 °C for 48 h in a humidified 5 % CO_2_ atmosphere.

The preparations were cultured in RPMI 1640 supplemented with 10 % FBS. After 48 h of incubation, 10 μg/ml of MTT [3-(4,5-dimethylthiazol-2-yl)-2,5 diphenyltetrazolium bromide]; (Sigma chemicals) was added to each well and incubated for 4 h at 37 °C in 5 % CO_2_. Following incubation, the supernatant from each well was removed and formazan crystals were solubilized by adding 100 μl dimethyl sulfoxide into each well.

The absorbance of each well was then determined at a wavelength of 540 nm, and the results expressed as a stimulation index (SI). SI was calculated as follows: SI = OD of stimulated culture/OD of unstimulated culture. All tests were performed in triplicate for each mouse.

### Cytokine secretion assay

One week after the third immunization, mononuclear cells from spleens of immunized mice at a concentration of 2 × 10^6^ cells per well were incubated in 24-well plates for 3 days in phenol red-free RPMI 1640 supplemented with 10 % FBS, 2 mM L-glutamine, 25 mM HEPES and 0.1 % penicillin/streptomycin, and pulsed with E7-specific H-2Db CTL epitope at a concentration of 1 μg/ml at 37° in 5 % CO_2_.

The supernatants were collected and assayed for the presence of IFN- γ, IL-4 and IL-12 using commercially available sandwich-based ELISA kits (eBioscience, San Diego, USA) following the manufacturer’s instruction. All tests were performed in triplicate for each mouse.

### Blockade of IL-18

One day prior to all of the vaccinations, Tc-1-treated mice (*n* = 3) were injected intra-peritoneally (i.p) with 100 mg of anti-mouse IL-18 clone 93–10 (R&D Systems) after reconstitution in PBS.

The control groups received an isotype matched IgG control reconstituted and injected in a similar fashion. One week following the final blockade and vaccine administration (4 weeks post tumor challenge), E7-specific IFN- γ response was determined (as described in cytokine secretion assay). The tumor volume was also monitored up to 6 weeks after tumor challenge.

### Statistical analysis

Lymphocyte proliferation, CTL and cytokine assay were analyzed by a one-way ANOVA. Significant differences of tumor growth on given days were assessed by Student’s *t*-test. Differences were considered statistically significant when *P value* < 0.05. All tests were performed in triplicate and all data are expressed as mean ± SD.

To compare results between the different groups, a one-way ANOVA was used. The statistical software SPSS version 16.0 was utilized for statistical analyses. Differences were considered statistically significant when *p* < 0.05.

## Results

### Cytotoxicity assay

Groups of C57BL/6 mice were immunized three times at one week intervals with E7 DNA vaccine with or without adjuvants of α-GalCer, MPL or combinations of α-GalCer and MPL. Non-radioactive cytotoxicity assay was performed to estimate the specific CTL cytolysis induced by various vaccines, in which the re-stimulated splenocytes as the effector cells were incubated with EL4 target cells pulsed with E7-specific H-2Db CTL epitope. Cytolytic activities are given as the mean percentages of specific lysis from three mice at effector: target ratio of 50:1 (50:1 E/T), with maximal CD8^+^ cytotoxic responses to EL4 target cells.

**Fig. 1:**
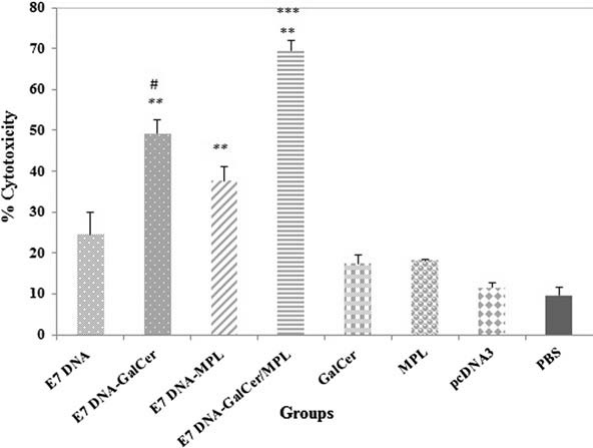
Legend. Analysis of the cytotoxic activity induced by E7 DNA vaccine plus α-GalCer and MPL combination. CTL activity of the lymphocytes from immunized mice (three mice per group) was measured at 50:1 E/T ratio by LDH release assay kit as described in Material and Methods section. Specific lysis of target cells are shown with nonspecific background lysis subtracted. LDH release was expressed as percent cytotoxicity ± S.D. *** Indicates statistically significant difference between E7 DNA vaccine in combination with α-GalCer and MPL group as determined by one-way ANOVA (*P* < 0.001) with other groups. ** shows the statistical significant differences between E7 DNA vaccine plus α-GalCer and E7 DNA vaccine plus MPL treatments than the E7 DNA vaccine alone group (*P* < 0.01). # shows the statistical significant differences between E7 DNA vaccine plus α-GalCer treatment than the E7 DNA vaccine plus MPL group (*P* < 0.05)

As shown in Fig. 1, injection of adjuvant alone and pcDNA3 induced very low antigen-specific CTL responses. In contrast, immunization with E7 DNA vaccine in association with α-GalCer and MPL induced significant cytolytic effects on target cells as compared with mice that immunized with the E7 DNA vaccine alone (*P* < 0.01). The cytolytic activity from mice vaccinated with E7 DNA vaccine plus adjuvant combination was at least 2 fold higher than those of E7 DNA vaccine alone. Furthermore, E7 DNA vaccine plus combination of α-GalCer-MPL showed the highest cytotoxic capacity (69.6 ± 2.3) of all the groups examined (*P* < 0.001). Notably, T lymphocytes from the mice vaccinated with the E7 DNA vaccine plus α-GalCer killed 49.2 % ±3.6 of target cells, which was significantly higher than that of the cells from E7 DNA vaccine plus MPL injected mice (37 % ±3.5, *P* < 0.05).

### Lymphocyte proliferation assay

Mice were immunized three times with the indicated amount of immunogens at one-week intervals: E7 DNA vaccine, E7 DNA vaccine plus α-GalCer, E7 DNA vaccine plus MPL, E7 DNA vaccine plus α-GalCer and MPL combination, MPL, α-GalCer, pcDNA3, vehicle or PBS. One week after the third immunization, the splenocytes from the immunized mice were harvested and re-stimulated in vitro with E7 49–57 for the lymphocyte proliferation assay.

**Fig. 2:**
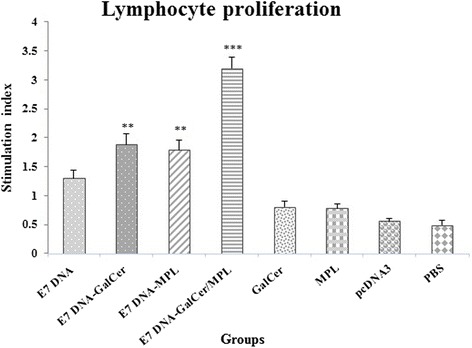
Legend. Lymphocyte proliferation levels after in vitro stimulation with HPV-16 E749–57 epitopes. The mice were injected subcutaneously thrice at 7-day intervals with DNA vaccine plus different adjuvant combinations. One week after final immunization, spleens of individual mice (three per group) were removed and lymphocyte proliferation was evaluated with MTT method. Values are the mean ± S.D. of the mean for the experiments. *** Indicates statistically significant difference between the E7 DNA vaccine in combination with α-GalCer and MPL group as determined by one-way ANOVA (*P* < 0.001) with other groups. ** shows the statistical significant differences between E7 DNA vaccine plus α-GalCer and E7 DNA vaccine plus MPL treatments than the E7 DNA vaccine alone group (*P* < 0.05)

As shown in Fig. 2, mice immunized with E7 DNA vaccine alone had low lymphocyte proliferation response (1.3 ± 0.14). In contrast, the in vitro re-stimulation with E749–57 led to significantly enhanced Ag-specific proliferation in the splenocytes isolated from mice immunized with E7 DNA vaccine plus α-GalCer and E7 DNA vaccine plus MPL (*P* < 0.05). In accordance with the CTL assay, splenocytes from mice inoculated with DNA vaccine plus α-GalCer and MPL combination showed evidently the greatest level of T lymphocyte proliferation upon in vitro stimulation with the E7 49–57 epitope with a mean SI value of 3.25 ± 0.19 (*P* < 0.001). However, there were no statistically significant differences in Ag-specific lymphocyte proliferation between α-GalCer and MPL adjuvanted groups (*P* = 0.058). As expected, very little proliferation was detected from mice immunized with MPL alone, α-GalCer alone, pcDNA3, vehicle or PBS.

### Cytokine secretion assay

**Fig. 3:**
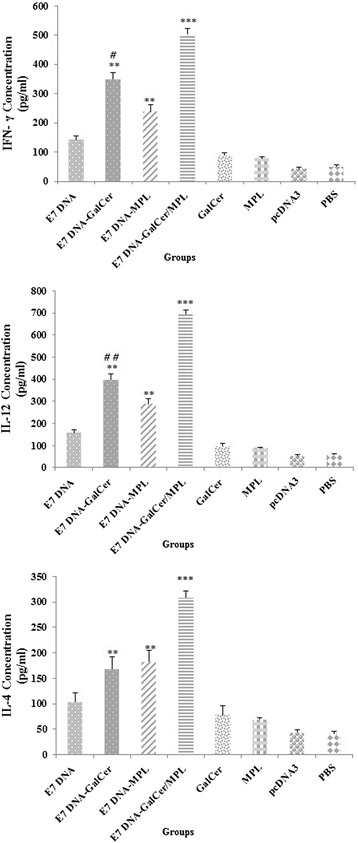
Legend. Concentration of IFN- γ (**a**), IL-12 (**b**) and IL-4 (**c**) in supernatant following stimulation of cultured splenocytes with E749–57 epitopes. Data presented as means ± S.D. for three mice per group. *** Indicates statistically significant difference (*P* < 0.001) between E7 DNA vaccine in combination with α-GalCer and MPL group as determined by one-way ANOVA (*P* < 0.001) with other groups (**a**, **b**, **c**). ** shows the statistical significant differences between E7 DNA vaccine plus α-GalCer and E7 DNA vaccine plus MPL treatments than the E7 DNA vaccine alone group (*P* < 0.01). **a**, **b** # (*P* < 0.05) and ## (*P* < 0.001) shows the statistical significant differences between E7 DNA vaccine plus α-GalCer treatment than the E7 DNA vaccine plus MPL group

In light of the high cellular immune response revealed by the CTL and lymphocyte proliferation assays, cytokine assay were performed to provide a more holistic profile of the cellular immunity induced by combination of α-GalCer and MPL as a DNA vaccine adjuvant. As shown in Fig. 3a-c, injection of MPL, α-GalCer, pcDNA3, vehicle or PBS did not induce any significant antigen-specific IFN- γ, IL-4 and IL-12 secretion. The production of these cytokines following the E7 DNA vaccine plus α-GalCer and E7 DNA vaccine plus MPL was significantly higher than in mice that received the unadjuvanted DNA vaccine (*P* < 0.01). Consistent with the CTL and lymphocyte proliferation assays, mice that received the DNA vaccine with both α-GalCer and MPL adjuvants exhibited substantially increased IFN- γ, IL-12 and IL-4 responses as compared with mice that received the E7 DNA vaccine plus MPL and E7 DNA vaccine plus α-GalCer (*P* < 0.01). Meanwhile, mice immunized with the E7 DNA vaccine plus α-GalCer showed significantly higher levels of IFN-$\gamma$ (*P* < 0.05) and IL-12 (*P* < 0.01) than those immunized with the E7 DNA vaccine plus MPL(*P* < 0.05); but no significant differences were observed in IL-4 production (*P* = 0.061). Thus, the immunological assessments showed that DNA vaccine adjuvanted with the combination of α-GalCer and MPL resulted in a synergistic enhancement of DNA vaccine– elicited cellular immune responses.

### In vivo tumor treatment experiment

To investigate whether α-GalCer and MPL adjuvants could induce regression and therapeutic effect against preexisting TC-1 tumors in DNA vaccinated mice, E7-expressing TC-1 tumor cells were injected into C57BL/6 mice at a dose of 2 × 10^5^ TC-1 cells in the right flank. One week later, C57BL/6 mice were immunized thrice with the E7 DNA vaccine with or without adjuvants, and monitored for tumor volume up to 6 weeks after the tumor challenge.

**Fig. 4:**
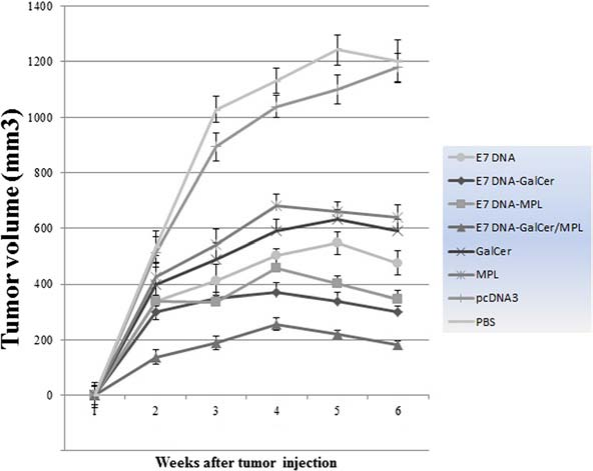
Legend. In vivo antitumor effects generated by treatment with E7 DNA vaccine plus α-GalCer, E7 DNA vaccine plus MPL, E7 DNA vaccine plus α-GalCer and MPL combination. C57BL/6 mice were inoculated with 2 × 10^5^ TC-1 tumor cells subcutaneously. Mice were then treated with DNA vaccine plus different adjuvant combinations as described in Materials and Methods. Mice were monitored for tumor growth by measuring diameters with calipers twice a week. Line and scatter plot graphs depicting the tumor volume (in mm3) are presented. The data presented are a representation of three independent experiments

As shown in Fig. 4, Tumors grew rapidly in mice inoculated with MPL, α-GalCer, pcDNA3, vehicle or PBS, whereas tumor growth was significantly retarded in mice that received DNA vaccine expressing E7 protein with or without adjuvants. The average tumor volume in the adjuvanted DNA vaccine and unadjuvanted DNA vaccine groups was significantly lower than that in the MPL, α-GalCer, pcDNA3, vehicle or PBS groups (*P* < 0.001). The statistical analysis of average tumor volumes exhibited significant difference between adjuvanted DNA vaccine groups and E7 DNA group (*P* < 0.001). At 21 and 28 days after vaccination, the average tumor volumes of the E7 DNA vaccine plus α-GalCer and MPL combination animals were significantly less (*P* < 0.01) than in those vaccinated with the E7 DNA vaccine plus α-GalCer and E7 DNA vaccine plus MPL groups. Furthermore, significant difference in average tumor volumes was detected between the E7 DNA vaccine plus α-GalCer -treated animals versus those treated with the E7 DNA vaccine plus MPL (*P* < 0.05). Similar results were obtained in three independent experiments with six mice per group.

### Effect of IL-18 blockade on Ag- specific IFN- γ secretion

**Fig. 5:**
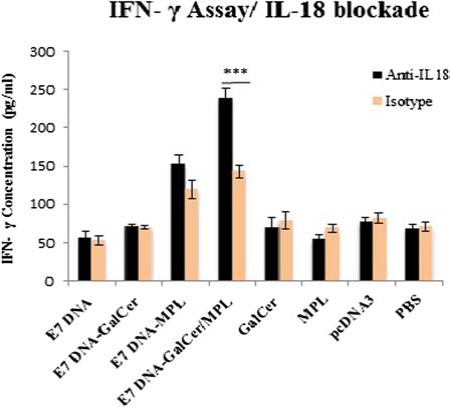
Legend. Anti-IL-18 blockade of TC-1-transplanted C57BL/6 mice during E7 DNA vaccination in combination with α-GalCer and MPL reduced the IFN- γ secretion. One week following the final blockade and vaccine administration, splenocytes were isolated and restimulated with E749–57 epitopes as described in Materials and Methods. The production of IFN- γ was measured from the supernatants of these restimulations by ELISA. Data are representative of three separate experiments. Data presented as means ± S.D. for three mice per group. Treatment groups were compared to the control group using one-way ANOVA. *** Indicates statistically significant difference (*P* < 0.001) between E7 DNA vaccine plus α-GalCer and MPL combination mice in the presence of anti-IL-18 mAbs over E7 DNA vaccine plus α-GalCer and MPL combination in the presence of isotype control

To determine whether blockade of IL-18 signaling during DNA vaccine plus α-GalCer and MPL immunization could also induce a greater IFN- γ response, C57BL/6 mice were vaccinated as before with concomitant administration of anti-IL-18 mAb or isotype control mAb 1 day prior to all of the vaccinations and for 3 weeks. One week following the final blockade and vaccine administration (4 weeks post tumor challenge), we determined the E7-specific IFN- γ response (Fig. 5). No significant increase in the levels of IFN-γ from E7-restimulated splenocytes was observed following DNA vaccination of C57BL/6 mice in the presence of anti-IL-18 mAbs, as compared to DNA vaccination in the presence of isotype control mAbs. However, mice immunized with E7 DNA vaccine plus α-GalCer and MPL combination in the presence of anti-IL-18 showed significant reduction in E7-specific IFN-γ response over E7 DNA vaccine plus α-GalCer and MPL combination group in the presence of isotype control mAbs (*P* < 0.001). Furthermore, E7 DNA vaccine combined with individual adjuvants in the presence of anti-IL-18 did not exhibit significant E7-specific IFN- γ decrease over mice immunized with E7 DNA vaccine combined with individual adjuvants in the presence of isotype control mAbs.

These data suggest that blockade of IL-18 during E7 DNA vaccine in combination with α-GalCer and MPL reduces IFN- γ secretion than E7 DNA vaccine combined with individual adjuvants.

### Effect of IL-18 blockade on therapeutic effect against TC-1 tumor

We determined whether abrogation of IL-18 signaling during DNA vaccine plus α-GalCer and MPL immunization only would lead to reduced therapeutic effect against TC-1 tumor. This would show whether IL-18 has a regulatory role specifically at the level of initial DNA vaccination, as has been shown in other immunization models (38–42). To do this we administered anti-mouse IL-18 mAb, or isotype control mAb, to C57BL/6 mice during vaccination, one day prior to all of the vaccinations, for 3 weeks and the tumor volume was monitored up to 6 weeks after the tumor challenge.

**Fig. 6:**
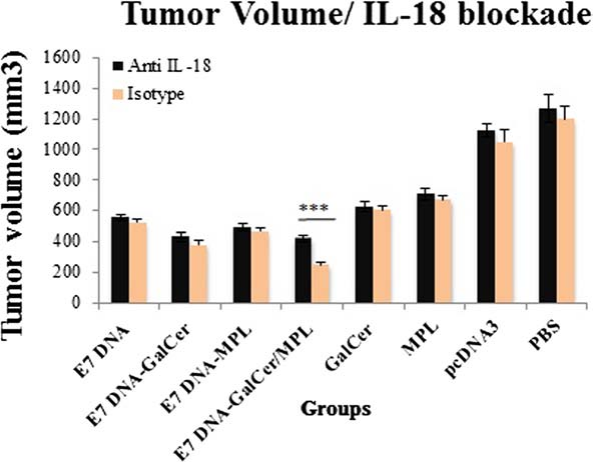
Legend. Anti-IL-18 mAb treatment during DNA vaccination decreases therapeutic effects against TC-1 tumor in C57BL/6 mice. One day prior to all of the vaccinations, Tc-1-treated mice (*n* = 3) were injected intra-peritoneally (i.p) with 100 mg of anti-mouse IL-18 or isotype control mAb. One week following the final blockade and vaccine administration, the tumor volume was monitored up to 6 weeks after the tumor challenge. Line and scatter plot graphs depicting the tumor volume (in mm3) are presented. The data presented are a representation of three independent experiments. Bar graph (Mean ± SD) showing average tumor volume on the day of 28. *** Indicates statistically significant difference (*P* < 0.001) between E7 DNA vaccine plus α-GalCer and MPL combination mice in the presence of anti-IL-18 mAbs over E7 DNA vaccine plus α-GalCer and MPL combination in the presence of isotype control

One week after last treatment, although E7 DNA vaccine plus α-GalCer and MPL combination mice in the presence of isotype control had significantly greater protection compared to DNA vaccination in the presence of isotype control mAbs, there was no additional tumor volume growth observed in E7 DNA vaccine plus α-GalCer and MPL combination mice in the presence of anti-IL-18 mAbs (data not shown). However by week 4, E7 DNA vaccine plus α-GalCer and MPL combination mice in the presence of anti-IL-18 mAbs showed an increase in tumor volume over E7 DNA vaccine plus α-GalCer and MPL combination in the presence of isotype control (Fig. 6). Additionally, mice immunized with E7 DNA vaccine combined with individual adjuvants in the presence of isotype control mice did not reveal further protection against tumor over E7 DNA vaccine combined with individual adjuvants in the presence of anti-IL-18 mAbs. These data suggest that blockade of IL-18 at the time of administering E7 DNA vaccine plus α-GalCer and MPL combination decreases the vaccine-driven antitumor response against TC-1 tumor as compared with other treatments.

## Discussion

The uses of adjuvants and relevant antigen targets in vaccine development have been an important focus of improving vaccine responses. The growing number of adjuvants can be used individually or in combination to maximize effects. However, there have been few reports addressing the potential use of adjuvants together with DNA vaccines. In this study, we examine the use of α-GalCer and MPL as a combined adjuvant for HPV-16 E7 DNA-based vaccination strategy. The results demonstrated that HPV-16 E7 DNA-induced E7-specific cellular immune responses and protection of animals from HPV 16 E7-associated tumor growth can be enhanced with α-GalCer and MPL adjuvant combination, as evidenced by significant enhanced lymphocyte proliferation, IFN-γ, Il-12, IL-4, cytotoxicity and in vivo anti-tumor effects, compared with control groups. However, injection with either E7 or individual adjuvants failed to show significant protection from tumor growth. These suggest that both E7 and α-GalCer -MPL adjuvant combination are required for therapeutic vaccine efficacies in this tumor model system.

It has been shown that that administration of iNKT cell ligands induces phenotypic and functional maturation of DCs leading to the priming of CD4 and CD8 effector T cells to antigens in mice. Furthermore, α-GalCer can stimulate NK activity and cytokine production by NKT cells and exhibits potent cytolytic activity in vivo [26]*.* Invariant Natural killer T cell (iNKT cells) are a subset of T cells that recognize glycolipid α-GalCer antigen bound by the major histocompatibility complex (MHC)-class-I-related protein CD1d, a non-polymorphic non-classical MHC class I molecule [27].

In agreement with our antitumor findings, it has been stated that administration of α-GalCer with DNA vaccines showed adjuvant effects against tumors. A DNA vaccine expressing HPV-16 E7 in combination with α-GalCer induced a significant E7-specific CD8 + T cell response in immunized mice through stimulating maturation of DCs. In fact, priming with a DNA vaccine in the presence of α-GalCer and boosting with E7-pulsed DC led to a significant enhancement of E7-specific CD8(+) effector and memory T-cells as well as significantly improved therapeutic effects against an E7-expressing tumor model (TC-1) in vaccinated mice [28]. It was also demonstrated that α-GalCer had adjuvant activity on HIV-1 DNA vaccines after administration at priming, leading to the enhancement of both antigen-specific cellular and humoral responses [29]. Guillonneau et al. demonstrated that giving alpha-GalCer with an inactivated influenza A virus subcutaneously improved protective efficacy of inactivated influenza A virus (IAV) [30].

α-GalCer has also been shown to enhance the anti-tumor activity in mice when administered in combination with various types of vaccines [9, 31–33]. Choi el al showed that treatment of the transgenic mice with ovarian tumor cell-based vaccines combined with adjuvant α-GalCer led to prolonged survival as well as increased numbers of tumor-specific CD8+ T cells [34]. Therefore, α-GalCer represents an important adjuvant for improving the efficacy of tumor cell-based vaccines to treat ovarian cancer.

Although the adjuvant properties of α-GalCer have been demonstrated in many vaccine animal models. However, limitations of single-adjuvant vaccine formulations are driving the need to explore combination adjuvants. For this reason investigators are exploring the potential of using formulations with multiple adjuvants in a vaccine. Several adjuvant combinations have been tested. Some adjuvant combinations even show a synergistic response following the co-administration of adjuvants: the effect of the two adjuvants combined is more beneficial than the sum of the effect of each adjuvant [35].

Because the DCs maturation after presentation of α-GalCer to NKT cells seems to operate independently of TLR adaptor protein MyD88 [26], therefore recent studies have highlighted the potential for synergizing the interactions between TLR ligands and iNKT cell activation in the design of effective vaccine adjuvants.

Hermans et al. provided evidence for cooperation between the adjuvant activities of α-GalCer and MPL, which binds with TLR4. It was showed that the simultaneous administration of iNKT cell ligand α-GalCer and MPL had a synergistic effect on the induction of CD8+ T-cell and antibody responses to OVA in wild-type animals compared with animals treated with the ligands individually. Antigen-specific CD8(+) T cell responses induced in the presence of the α-GalCer and MPL showed improved effector and proliferation function, than those induced with either ligand alone[36], the findings are in agreement with our current results. In another study, it was described that DC maturation induced by a TLR ligand was also enhanced by *i*NKT cell activity. Exposure of APCs to both TLR-mediated and *i*NKT cell–mediated signals stimulated significantly greater DC-induced T-cell immunity than exposure to either stimulus alone [33].

To understand whether IL-18 blockage following DNA vaccine plus α-GalCer-MPL combination limited vaccine efficacy in terms of the IFN- γ generated, and most importantly whether this would limit therapeutic effects against TC-1 tumor, C57BL/6 mice were administrated with anti-mouse IL-18 Ab, or isotype control mAb, 1 day prior to all of the vaccinations, and subsequently analyzed by the IFN-γ secretion and tumor volume.

We found that blockade of IL-18 signaling in E7 DNA vaccine plus α-GalCer and MPL combination mice reduced E7-specific IFN-γ, and that this treatment decreased the protection against tumor growth compared with isotype control mAb.

Our findings are in agreement with previously published studies demonstrating stimulatory role of IL-18 in the antitumor immune response via induction of IFN- γ production from NK and CD4+ T cells [37].

IL-18, IFN- γ-inducing factor, acts synergistically with IL-12 in inducing IFN- γ synthesis by a NKT and NK cells to kill target cells in a Fas ligand-dependent manner [38]. IFN- γ has a significant role in enhancing cell-mediated antitumor immunity by increasing tumor immunogenicity through enhancing antigen presentation as well as prompting antigen-presenting cells, NK cells, CTLs and Th1 T cells [39].

Furthermore, several studies have shown the antitumor efficacy of IL-18 in animal tumor models. For example, IL-18 exerted antitumor immunity by activating NK cells and establishing cytotoxic CD4+ T cells [37]. In support of the latter possibility, blockade of IL-18 presented marked reduction in IFN-γ production after HPV-16 E7 re-stimulation. In addition, absence of the cytokine led to worsening of tumor growth.

## Conclusions

Taken together, our study shows that co-administration α-GalCer and MPL with E7 DNA could significantly reduce tumor volume and eradicate the established E7-expressing tumors. The combination adjuvants might be able to employ low doses of iNKT cell antigens, thus avoiding some of the obstacles associated with vaccines that require high doses of iNKT cell antigens. Additionally, the rational combination of the adjuvants is likely to offer opportunities for the development of therapeutic vaccines for therapy of cervical cancer.

## Abbreviations

HPV: Human papilloma virus
PHA: Phytohemaglotinin
APC: antigen-presenting cell
CTL: cytolytic T lymphocyte
DC: Dendritic cells
IFN- γ: interferon γ
IL-4: interleukin 4
TNF: Tumor necrosis factor
TGF-β: Transforming growth factor beta
PVDF: polyvinylidene difluoride membranes
LDH: lactate dehydrogenase
MTT: 3[4,5-dimethylthiazol-2-ll]-2,5-diphenyltetrazolium bromide; thiazolyl-blue
DMSO: Dimethyl sulfoxide
OD: optical density
FBS: Fetal bovine serum
RPMI: 1640 Roswell Park Memorial Institute (name of the medium)
Th: T helper
PAMP: Pathogen-associated molecular patterns
MPL: Monophosphoryl lipid A
TLR: Toll-like receptors
